# Supplementary figures and images for: PI-Plat: a high-resolution image-based 3D reconstruction method to estimate growth dynamics of rice inflorescence traits
Source: Plant Methods. 2019 Dec 27;15:162. doi: 10.1186/s13007-019-0545-2 (PMC6933716; doi:10.1186/s13007-019-0545-2)

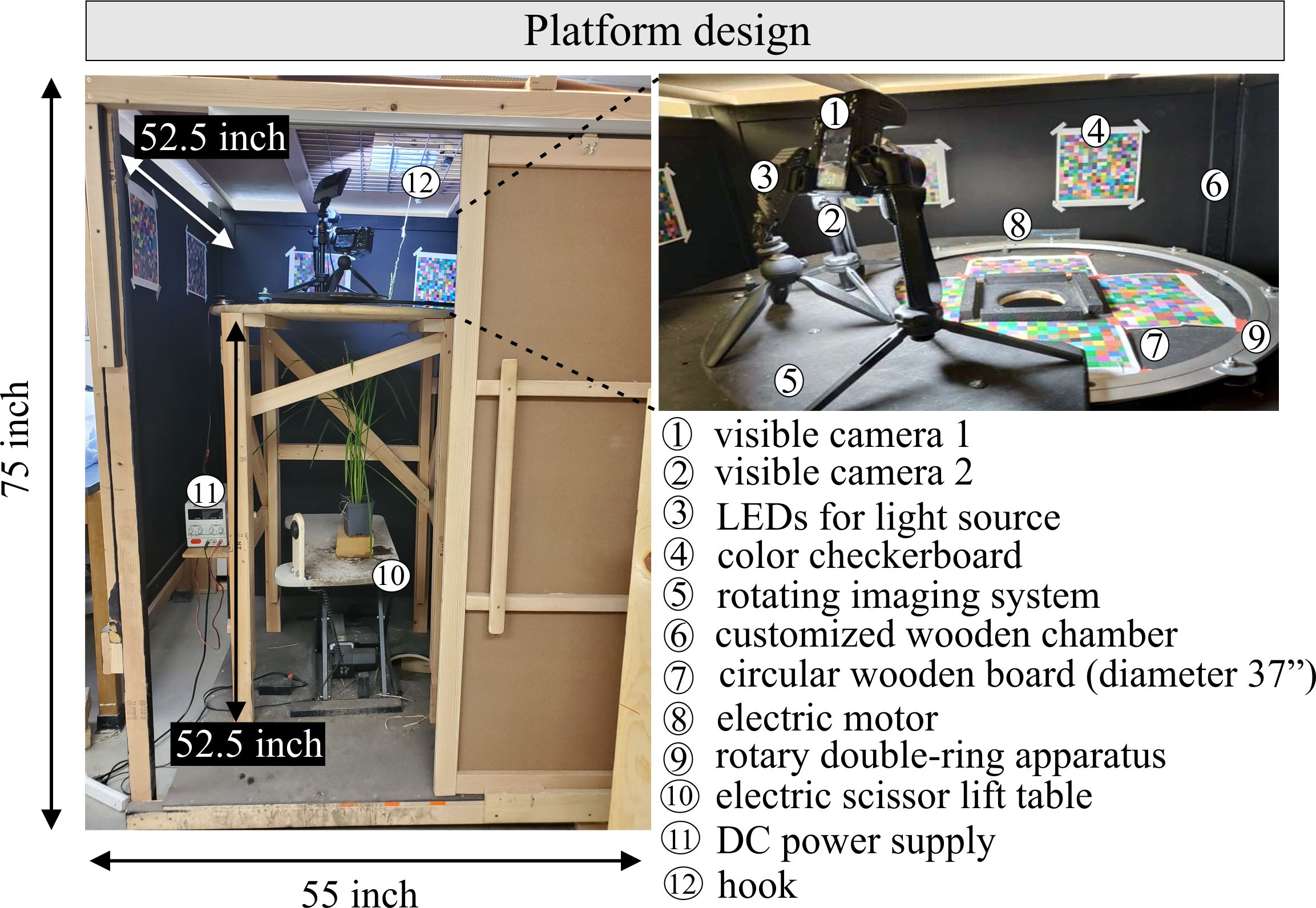

Supplement: Supplementary file 2 — Additional file 2. PI-Plat and its components. [file 13007_2019_545_MOESM2_ESM.jpg]

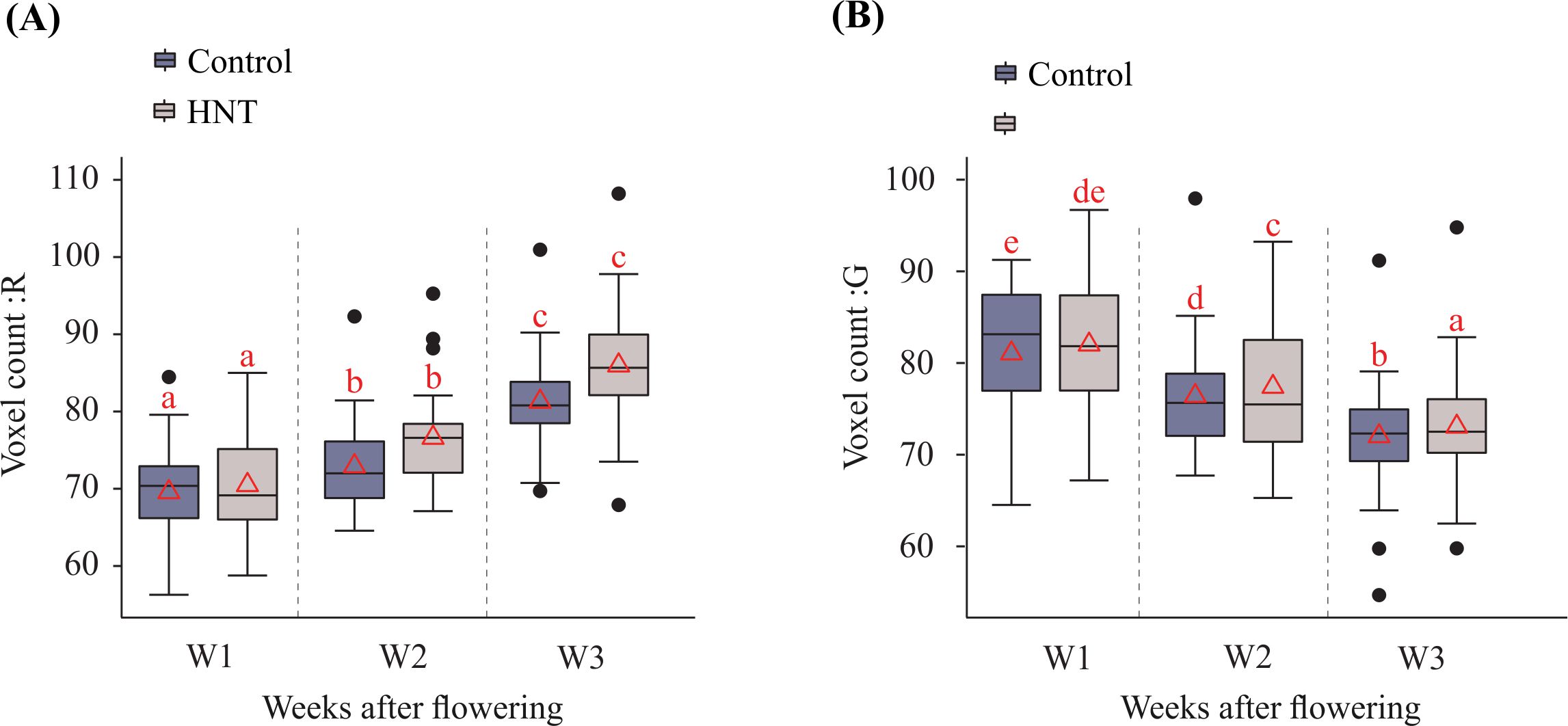

Supplement: Supplementary file 6 — Additional file 6. Average intensities of (A) red and (B) green channels from all genotypes for a respective treatment (control and HNT) and time-point (week 1, 2, and 3). [file 13007_2019_545_MOESM6_ESM.jpg]
